# Normal gray matter volumes in women recovered from anorexia nervosa: a voxel-based morphometry study

**DOI:** 10.1186/s12888-016-0856-z

**Published:** 2016-05-13

**Authors:** Lasse Bang, Øyvind Rø, Tor Endestad

**Affiliations:** Regional Department for Eating Disorders, Division of Mental Health and Addiction, Oslo University Hospital, P.O. Box 4956, Nydalen, 0424 Oslo, Norway; Division of Mental Health and Addiction, Institute of Clinical Medicine, University of Oslo, P.O. Box 1171, Blindern, 0318 Oslo, Norway; Institute of Psychology, University of Oslo, P.O. Box 1094, Blindern, 0317 Oslo, Norway

**Keywords:** Anorexia nervosa, Voxel based morphometry, Magnetic resonance imaging, Structural brain alterations, Gray matter

## Abstract

**Background:**

Anorexia nervosa (AN) has consistently been associated with reduced gray (GM) and white matter (WM) brain volumes. It is unclear whether GM alterations are present following recovery from AN, as previous findings are inconsistent. The aim of the present study was to determine if women recovered from AN exhibit reduced global or regional GM volumes.

**Methods:**

Global GM and WM, as well as regional GM volumes, were investigated in 22 women recovered from AN and 22 age-matched healthy controls using magnetic resonance imaging. Women were considered recovered if they had maintained a body mass index above 18.0 and had not engaged in binge eating, purging, or restrictive eating behaviors during the past year.

**Results:**

There were no significant differences between recovered AN women and healthy controls in terms of GM and WM volumes. There were also no significant differences between restricting and binging-purging AN subtypes. Lowest lifetime weight was positively correlated with regional GM volumes in the precuneus and insula.

**Conclusions:**

The present study showed that regional GM and global GM and WM volumes were similar for women long-term recovered from AN and age-matched healthy controls. Further research is needed to determine the extent to which illness severity affect regional GM volumes.

## Background

Anorexia nervosa (AN) has consistently been associated with reduced global gray (GM) and white matter (WM) brain volumes, alongside enlarged cerebrospinal fluid (CSF) cavities [1, 2]. Furthermore, studies have shown focal GM reductions in widespread subcortical and neocortical regions in patients with AN [2].

Considerable research effort has been devoted to determine whether the structural brain alterations persist following weight-restoration and recovery from AN, but extant findings are equivocal. Longitudinal studies of AN patients have shown normalization of WM but persistent loss of GM at follow-up [3–5]. Normal WM, but reduced GM volumes have also been reported in cross-sectional studies of individuals recovered from AN [6–9], with one study even reporting *increased* GM volumes in several regions [10]. This suggests that while WM normalize, GM reductions persist following weight-restoration and recovery.

In contrast, several studies have failed to detect any GM alterations in recovered AN patients [11–14], indicating that GM reductions observed during the acute phase of the illness are reversible, and thus secondary to emaciation. The inconsistent findings regarding the presence of GM volume reduction in recovered patients may be due to heterogeneity in study design, for example, differences in the operationalization or duration of recovery, or variations in the length of follow-up interval in longitudinal studies. Also, several studies are limited by small sample sizes. In sum, it remains unclear if individuals recovered from AN are characterized by reduced GM mass, and more studies are warranted [2].

To increase our understanding of the neurobiological underpinnings of AN, it is important to ascertain the presence of GM alterations in recovered AN individuals.

GM reductions following recovery may reflect a trait-like neurobiological risk factor for developing AN, and thus be important in the etiology of the disorder. Alternatively, such reductions may reflect irreversible changes subsequent to emaciation. In the present study, we used magnetic resonance imaging (MRI) to investigate global cerebral mass and regional GM volumes in women recovered from AN.

## Methods

### Participants

We recruited 22 adult women recovered from AN (RAN) and 22 age-matched, healthy control women via user-organizations for eating disorders, printed flyers, and online forums. Current and lifetime DSM-IV diagnoses were evaluated with the Structured Clinical Interview for DSM-IV Axis I Disorders (SCID-I/P, [15]). All women in the RAN group had a lifetime history of AN according to the DSM-IV criteria [16], excluding the amenorrhea criterion. Women were considered recovered if they had maintained a body mass index (BMI, kg/m^2^) above 18.0 and had neither engaged in binging and purging behavior, nor severely restricted food intake for the past year. Exclusion criteria for these women included: lifetime history of a psychotic disorder, substance abuse or dependence, or the presence of any Axis I disorder the past year.

Control women had no lifetime history of any Axis-I disorder and took no psychoactive medications. Furthermore, we excluded control women who reported binging and purging behavior, excessive and compulsive exercising, severely restricted food-intake, or a body mass index below 18.0 for the past 12 months. Women in both groups were excluded if they reported any major medical illnesses, history of severe head trauma, or any MRI-contraindications.

Three in the RAN group were using psychoactive medications, but results did not change when these were excluded, so they were included in the final analyses. This study was approved by the Regional Ethics Committee in Norway, and all participants provided written informed consent prior to onset of the study.

### Behavioral measures

To characterize the recovery status of RAN women, all participants completed the Spielberger State-Trait Anxiety Inventory (STAI; [17]), Beck Depression Inventory-II (BDI; [18]), and Eating Disorder Examination-Questionnaire (EDE-Q; [19]). Participants were also weighed in order to calculate their current BMI.

### MRI image acquisition

Images were acquired with a three Tesla Achieva MRI scanner (Philips, Eindhoven). High-resolution structural images were acquired using a T1-weighted multi-shot turbo-field-echo sequence (TR/TE = 6.7/3.1 milliseconds, flip angle = 8°, FOV = 256 × 256 mm, matrix = 256 × 213), recording 170 sagittal slices covering the whole brain (voxel size = 1.0 × 1.2 × 1.2).

### MRI image analysis

#### Preprocessing of MRI images

Images were preprocessed using the Voxel-Based Morphometry 8 (VBM8) toolbox (http://dbm.neuro.uni-jena.de/vbm8), an extension of the Statistical Parametric Mapping 8 (SPM8) software (http://www.fil.ion.ucl.ac.uk/spm). This involved segmenting images into GM, WM, and CSF. The GM images were spatially normalized and bias-field corrected using high-dimensional diffeomorphic anatomical registration through exponentiated Lie (DARTEL) algebra [20]. The resulting modulated GM images (corrected for individual brain size) were then smoothed with a 10 mm full-width half-maximum kernel.

#### Global tissue volumes

Raw global tissue volumes (GM, WM, CSF) were extracted using VBM8, and total intracranial volume (TIV) was calculated by adding these tissue volumes. To correct for individual brain size, GM, WM and CSF volumes were converted to fractions by dividing them by the TIV. These fractions were then submitted to two-sample t-tests, testing for differences between healthy controls and RAN individuals.

Additional t-tests were performed to investigate potential differences in TIV, GM, WM, and CSF between individuals with a history of restricting AN subtype and binging-purging AN subtype. Spearman *r*_*s*_ correlations were calculated between tissue fractions (GM, WM, CSF) and clinical characteristics (recovery duration, illness duration, AN onset, and lowest weight ever) in the RAN group, to characterize potential associations.

#### Regional gray matter volumes

The modulated and smoothed MRI images were submitted to a two-sample t-test in SPM8, to test for differences between healthy controls and RAN individuals. We first evaluated the presence of between-group effects within two a priori regions of interest (ROI), followed by a whole-brain analysis. Our ROIs consisted of the anterior cingulate cortex (ACC) and the supplementary motor area (SMA). These regions were chosen due to frequent reports of reduced GM volume in these areas in both the ill [6, 21–25] and recovered AN state [3, 6, 7], making them the most commonly reported loci of GM reduction in AN. The ACC was defined using a maximum probability atlas [26], available from www.brain-development.org. The SMA was defined by creating a sphere (radius = 15 mm) centered on the Montreal Neurological Institute (MNI) coordinates 4–6 59 (x, y, z; coordinates based on previous studies reporting SMA reduction).

A second t-test was performed, comparing individuals with a history of restricting subtype AN, and individuals with a history of binging-purging AN. As described above, we first explored the presence of between-group effects in the ACC and SMA, followed by a whole-brain analysis. Lastly, because previous studies have indicated that lowest lifetime weight [7, 8, 27] and illness duration [28] is associated with regional GM reductions in AN, an exploratory whole-brain multiple regression analysis was performed. Lowest lifetime weight and illness duration were entered as predictors of voxel-wise GM volumes in the RAN group. Age, current BMI, and GM fraction were entered as covariates. For significant associations, *R*^*2*^ for the peak-voxel within each cluster is reported. Furthermore, for all significant associations, we extracted the total value for all significant voxels within each cluster separately, using the get_totals script by Ged Ridgway (http://www0.cs.ucl.ac.uk/staff/g.ridgway/vbm/get_totals.m). These sum values were plotted against the predictor variables (lowest lifetime weight and illness duration). This was done to exclude the possibility of purely outlier-driven effects (which are not reported), and for illustrative purposes.

For the ROI analyses, we used small volume corrections, and voxels were considered significant if they survived a threshold of voxel-level *p* < .05 family-wise error-corrected. For all whole-brain analyses, two thresholds were applied to designate significant results: *p* < .05 family-wise error corrected for multiple comparisons, and *p* < .001 uncorrected for multiple comparisons with a cluster extent (*k*_*e*_) > 50 voxels.

## Results

### Participant characteristics

RAN and control women were of similar age, but BMI was significantly higher for the controls (see Table 1). Relative to controls, women recovered from AN scored higher on the STAI, BDI, and EDE-Q (see Table 1), indicating increased levels of anxiety, depression, and eating disorder psychopathology. All participants scored below the empirically established global EDE-Q clinical cut-off value of 2.5 [29]. Half of the recovered AN women (*n* = 11) had a history of AN binging-purging subtype, while the remaining half (*n* = 11) had a history of AN restricting subtype. Clinical characteristics of the RAN women are shown in Table 1. The majority of RAN women (*n* = 19, 86 %) reported that they received treatment during their period of AN.

**Table 1:** Participant characteristics and global tissue volumes in women recovered from anorexia nervosa versus healthy controls

	RAN (*n* = 22)	HC (*n* = 22)	Two-sample t-test		
Characteristic	Mean ± SD	Mean ± SD	*t (df)*	*P*	*d*
Age	27.32 ± 5.14	26.14 ± 4.64	0.80 (42)	.43	0.24
BMI (kg/m^2^)^a^	20.39 ± 1.66	21.85 ± 1.76	−2.70 (38)	.01	−0.85
BDI	6.36 ± 7.94	1.77 ± 2.69	2.57 (42)	.02	0.77
EDE-Q global score	0.84 ± 0.74	0.19 ± 0.17	4.04 (42)	<.001	1.21
STAI state score	32.14 ± 8.16	25.86 ± 5.21	3.04 (42)	.004	0.92
STAI state score	38.77 ± 11.48	28.36 ± 6.43	3.71 (42)	.001	1.12
GM volume (ml)	635.96 ± 52.03	664.39 ± 44.81	1.94 (42)	.06	−0.59
WM volume (ml)	474.31 ± 51.71	479.70 ± 39.19	0.39 (42)	.70	−0.12
CSF volume (ml)	224.82 ± 30.61	232.19 ± 21.40	0.93 (42)	.36	−0.28
TIV (ml)	1335.09 ± 115.62	1376.28 ± 87.11	1.34 (42)	.19	−0.40
GM fraction (GM divided by TIV)	0.477 ± 0.021	0.483 ± 0.015	1.06 (42)	.30	−0.33
WM fraction (WM divided by TIV)	0.355 ± 0.017	0.348 ± 0.014	−1.39 (42)	.17	0.45
CSF fraction (CSF divided by TIV)	0.168 ± 0.015	0.169 ± 0.012	0.17 (42)	.87	−0.07
Lowest lifetime weight^b^	71.84 ± 9.18 (range: 46–85)				
Lowest lifetime BMI (kg/m^2^)^c^	14.76 ± 1.83 (range: 10–17)				
Age of AN onset	17.36 ± 4.17 (range: 11–32)				
Duration of illness (months)^d^	32.86 ± 27.47 (range: 6–120)				
Duration of recovery (months)^d^	51.62 ± 42.70 (range: 12–192)				

*BDI* Beck depression inventory, *BMI* Body mass index, *CSF* Cerebrospinal fluid, *d* Cohen’s *d* effect size, *EDE-Q* Eating disorder examination-questionnaire, *GM* Gray matter, *HC* Healthy controls, *ml* milliliters, *RAN* Recovered anorexia nervosa, *STAI* State-trait anxiety inventory, *TIV* Total intracranial volume, *WM* White matter

^a^Data not available for three recovered anorexia nervosa women and one healthy control

^b^Expressed as percentage of ideal weight, taking into account height, age and gender

^c^Not adjusted for age

^d^Data not available for one recovered anorexia nervosa woman

### MRI results

#### Global tissue volumes

There were no differences in TIV, global GM, WM or CSF volume fractions between healthy controls and RAN women (see Table 1). Because the t-tests between controls and RAN women on WM and TIV were associated with low *p*-values (*p* = .17 and *p* = .19, respectively), we bootstrapped the mean difference between groups on these variables (1000 resamples of each group), to explore the accuracy of the estimated mean difference. Confidence intervals (95 %) for the mean difference were:-.014 - .002 for WM, and-18.48–95.93 for TIV. This further suggests that the mean difference in WM and TIV between groups is non-significant.

There were also no differences between individuals with a history of restricting AN subtype and binging-purging AN subtype in TIV (*t*[20] = 1.20, *p* = .243), GM fraction (*t*[20] = −0.07, *p* = .949), WM fraction (*t*[20] = 0.37, *p* = .719), or CSF fraction (*t*[20] = −0.32, *p* = .752). Within the RAN group, clinical characteristics (recovery duration, illness duration, AN onset, and lowest lifetime weight) did not show statistical significant associations with GM, WM or CSF volume fractions (*r*_*s*_, all *p* > .05).

#### Regional gray matter volumes

ROI and whole-brain VBM analyses revealed no significant regional differences in GM between healthy controls and RAN women. Including age, current BMI and GM fraction as covariates did not alter the results, although a trend-level difference (*p* < .005, uncorrected for multiple comparisons) was present in the middle frontal gyrus. ROI and whole-brain VBM analyses also failed to detect significant regional differences in GM between individuals with a history of restricting AN and individuals with a history of binging-purging AN.

**Fig. 1:**
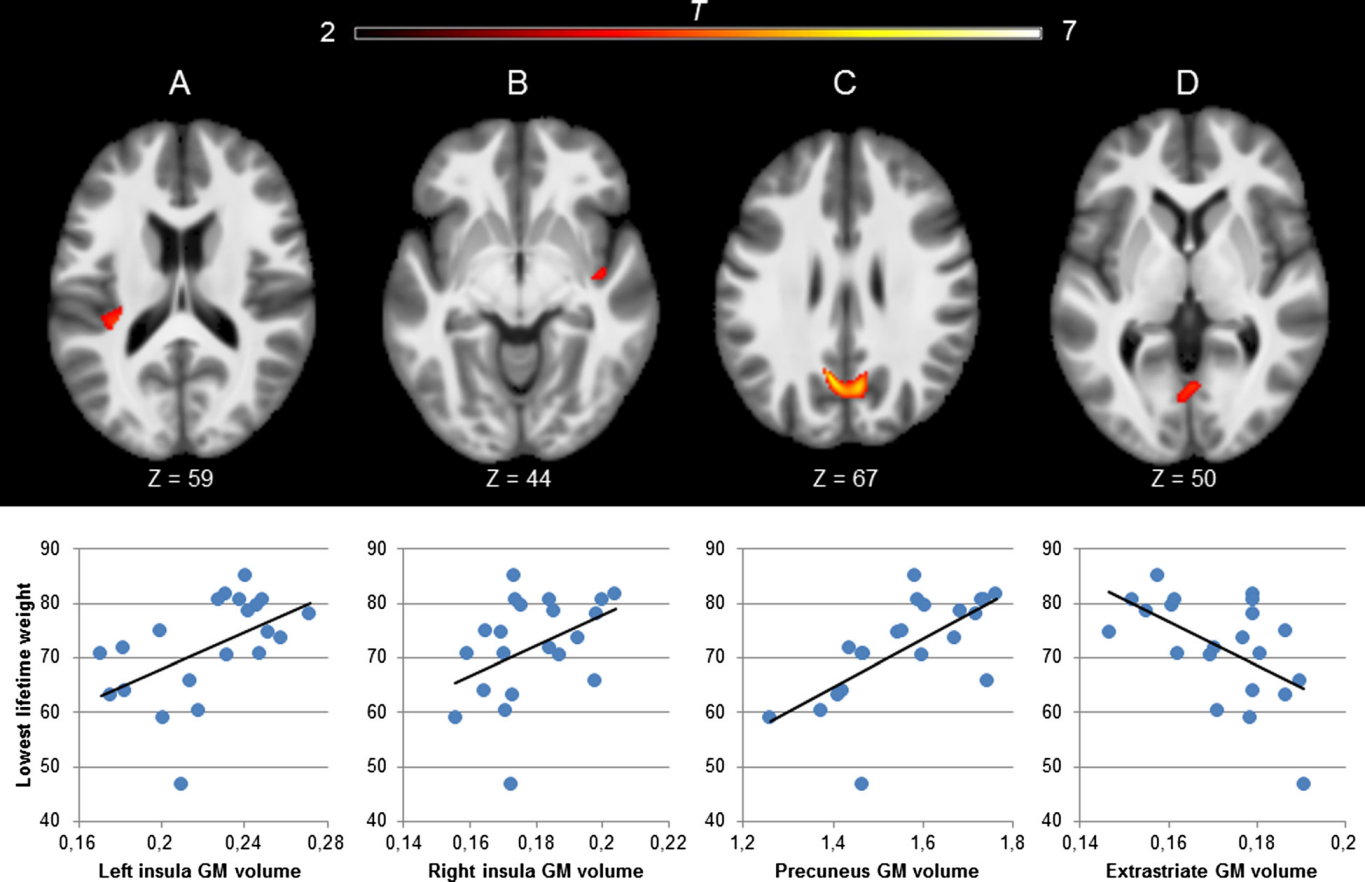
Results from multiple regression analysis showing regions that correlate with lowest lifetime weight (results shown at *p* < .001 uncorrected for multiple comparisons, *k*_*e*_ > 50). Activation maps are overlaid on a group average anatomical image (left side corresponds to left brain hemisphere). For illustrative purposes, the total value for all voxels within each cluster separately was calculated and plotted against lowest lifetime weight, and are shown below the corresponding activation maps. A: Left insula. B: Right insula. C: Precuneus. D: Extrastriate cortex

The whole-brain multiple regression analysis showed that lowest lifetime weight was positively correlated (*p* < .001 uncorrected for multiple comparisons) with regional GM volumes in the precuneus (MNI[x, y, z] = −5,−67, 30; *t*[15] = 6.39, *z* = 4.38, *k*_*e*_ = 724, peak-voxel *R*^*2*^ = .71), left insula (MNI[x, y, z] = −32,−33, 13; *t*[15] = 4.31, *z* = 3.42, *k*_*e*_ = 168, peak-voxel *R*^*2*^ = .34) and right insula (MNI[x, y, z] = 44,−4,−15; *t*[15] = 4.20, *z* = 3.36, *k*_*e*_ = 89, peak-voxel *R*^*2*^ = .53). Moreover, lowest lifetime weight was inversely correlated with a cluster in the extrastriate cortex (MNI[x, y, z] = 3,−67, 3; *t*[15] = 4.49, *z* = 3.52, *k*_*e*_ = 77, peak-voxel *R*^*2*^ = .56). These associations did not survive correction for multiple comparisons, and there were no significant associations between illness duration and regional GM volumes. For purely illustrative purposes, the total values for all voxels within each cluster separately were plotted against lowest lifetime weight, and are presented in Fig. 1.

## Discussion

In the present study, regional GM and global GM, WM, and CSF volumes were similar for women long-term recovered from AN and age-matched healthy controls. There were no cerebral tissue volume differences between individuals with a history of restricting AN subtype and individuals with a history of binging-purging AN subtype. This indicates that the failure to detect differences in tissue volumes between healthy controls and RAN women were not due to effects of AN subtype.

Our results support findings from previous studies, showing normal cerebral tissue volumes in individuals recovered from AN [11–14]. In a longitudinal study, Mainz and colleagues [13] showed that while adolescent AN patients had reduced GM volume when ill, these changes fully normalized at discharge. Moreover, the GM gain observed was positively correlated with weight and inversely correlated with cortisol levels, providing evidence that GM mass reverse in conjunction with hormonal and weight restoration. Wagner and colleagues [14] also found normal global and regional GM volumes in normal-weight women long-term recovered from AN.

These findings suggest the loss of GM in patients do not reflect atrophy or AN-related neurobiological risk factors, but rather temporary and reversible changes in brain microstructure. Although the mechanisms behind these changes remain unclear, they could be related to hormonal imbalances, for instance excessive cortisol levels [3, 13, 27]. Cell-shrinkage due to dehydration might also be a contributing factor, as dehydrated healthy individuals show transient reductions of GM and WM [30].

In contrast, others report persistent GM loss following recovery from AN [3–7, 10]. The conflicting results between studies showing GM reductions in recovered AN individuals and the present study may be attributed to differences in study design, for example sample size, inclusion criteria, and operationalization and duration of recovery. For example, Mühlau and colleagues [7] reported reduced global and regional GM volumes among recovered AN patients. However, liberal criteria for recovery were used, requiring only a minimum BMI ≥ 17.0 and regular menses for 6 months. One challenge is that there is no universal definition of recovery from AN [31]. Subsequently, there is great variability in how investigators define recovery, which hampers cross-study comparisons. In the present study, we emphasized weight-restoration and absence of pathological behaviors (e.g. binging, restrictive food-intake) over a 1-year period in our definition of recovery, similar to other studies [14]. Adequate weight restoration and ample duration of recovery may be crucial for brain normalization to occur. Indeed, longitudinal studies have shown that GM and WM volumes increase upon weight restoration [3, 5, 13, 32] and reports of persistent GM loss at follow-up may be due to short follow-up intervals [3, 5]. In our study, the duration of recovery was minimum 1 year, and on average 4 years, which may be sufficient for normalization of cerebral tissue.

One exception is the Friedrich et al. (2012) study, which reported reduced regional GM volumes in women who had been recovered for many years [6]. Our study failed to detect similar regional GM reductions, even though participants’ average duration of recovery and BMI was comparable between studies. The reasons for these conflicting results are unclear. However, it is worth noting that the sample size in the study by Friedrich et al. was small (*n* = 13).

In the present study, lowest lifetime weight was positively correlated with GM volumes in the precuneus and insula. This suggests that GM volumes in these areas are influenced by the severity of AN, even years after weight-restoration and recovery. Similar associations between lowest lifetime weight and GM volumes have been reported in a few previous studies [7, 27], but not specifically in the precuneus and insula. However, Joos and colleagues [8] reported that recovered women with a history of severe AN showed decreased GM volumes in the precuneus, and suggested that certain irreversible pathophysiological processes can occur when patients’ weight drops below a critical threshold. In a similar vein, there is some evidence to suggest that longer illness duration also exacerbates GM reductions in AN [25, 28]. Interestingly, Bär and colleagues [25] showed that illness duration was inversely correlated with GM volume in the precuneus among AN patients, suggesting this area of the brain is particularly susceptible to the effects of AN. However, no association between illness duration and regional GM volumes was found in the present study, and similar null-findings have been reported by others [10, 33].

The severity of AN may be an important factor when considering the reversibility of regional GM reductions. It is possible that certain subgroups of recovered AN patients, for instance those with a history of extreme emaciation or long illness duration, are characterized by persistent regional GM reductions following recovery. This could account for why the present study found significant associations between lowest lifetime weight and GM volumes in the RAN group, but no significant between-group differences in GM volume. The impact of the GM reductions incurred by severe AN are likely subtle, and not easily detected in case–control studies where there typically is considerable sample heterogeneity. These issues could partly be the source of contrasting evidence regarding the presence of GM reductions in recovered AN individuals. Selectively investigating individuals with a history of severe AN, as some have already done [8, 28], may be one fruitful avenue for future studies, to ensure sufficient power is achieved.

The present study also found a significant inverse association between lowest lifetime weight and GM volume in the extrastriate cortex. The direction of this relationship is puzzling considering previous research and the issues discussed above, and its nature is unclear. Of note, the cluster in the extrastriate cortex showing this association was small, and like the other significant associations, did not survive correction for multiple comparisons. It is important to acknowledge the possibility of spurious associations, particularly when results are uncorrected for multiple comparisons.

Limitations of the present study include the cross-sectional design, which precludes making longitudinal conclusions regarding the reversibility of cerebral mass. This design also entailed that lifetime AN diagnosis had to be established retrospectively. There was also considerable variability in the clinical characteristics of the RAN group, which might have influenced our results. Lastly, it is important to note that absence of evidence does not constitute evidence of absence [34], and the present study’s failure to detect regional GM alterations in AN does not imply that there is none. The sample size in the present study was modest, and it cannot be ruled out that small reductions in regional or global brain volumes in women recovered from AN would be evident with larger sample sizes. However, previous studies with sample sizes comparable to or smaller than our own have reported GM alterations in recovered AN individuals [6, 8–10], indicating the present study was sufficiently powered to detect true group differences of similar magnitude. As discussed, it is possible that GM reductions only are evident in certain subgroups of recovered AN individuals, for example those with severe clinical histories. Unless specifically recruiting such cases, moderate sample sizes might not be sufficiently powered to detect such patterns.

## Conclusions

To conclude, this study showed that regional GM and global GM and WM and volumes were similar for women long-term recovered from AN and age-matched healthy controls. Findings add to a growing evidence base suggesting that individuals recovered from AN have normal cerebral tissue volumes. However, this study did show significant associations between lowest lifetime weight and regional GM volumes which warrants further attention, as they suggest that subgroups of recovered AN individuals might exhibit regional GM reductions.

## Ethics and consent to participate

This study was approved by the Regional Committee for Medical and Health Research Ethics for Health Region South-East (REK Sør-Øst, reference nr. 2012/1386). All participants provided written informed consent prior to onset of the study.

## Consent to publish

Not applicable.

## Availability of data and materials

Due to issues regarding confidentiality and ethics, data cannot be shared.

## Abbreviations

ACC: anterior cingulate cortex
AN: anorexia nervosa
BDI: beck depression inventory
BMI: body mass index
CSF: cerebrospinal fluid
EDE-Q: eating disorder examination-questionnaire
GM: gray matter
MNI: montreal neurological institute
MRI: magnetic resonance imaging
RAN: recovered anorexia nervosa
ROI: region of interest
SMA: supplementary motor area
STAI: state-trait anxiety inventory
TIV: total intracranial volume
VBM: voxel-based morphometry
WM: white matter
